# Eco-friendly development of multi-functional textiles using rice straw extract

**DOI:** 10.1038/s41598-026-43684-5

**Published:** 2026-04-02

**Authors:** Marwa Abou-Taleb, Khlood S. Abdel Zaher, Salwa Mowafi, Galal A. M. Nawwar

**Affiliations:** 1 Proteinic and Man-made Fibres Department, Textile Research and Technology Institute, National Research Centre, Giza, Dokki, 12622 Egypt; 2 Green Chemistry Department, National Research Centre, Dokki, Giza, 12622 Egypt

**Keywords:** Natural dye, Phenolic compounds, Flavonoids, Wool, Nylon 6, Silk, Antioxidant, Chemistry, Environmental sciences, Materials science

## Abstract

Rice straw extract (RSE) is a significant source of phenolic compounds and flavonoids that can be used to functionalize textile fibers. Based on this finding, this work aims to study the potential of the extract from rice straw pulping liquor, as sustainable and multifunctional natural dye for simultaneous coloration and finishing of both natural (wool and silk) and synthetic (nylon 6) fabrics. Fabrics were treated by different concentrations of RSE (0.2, 0.4, 0.6, 0.8% wt/v) for 1 h. The effect of bath pH and temperature as well as atmospheric-plasma pretreatment on fabric affinity towards RSE was evaluated. RSE was characterized by Liquid chromatography-tandem mass spectrometry (LC/MS/MS), Fourier transforms infrared spectroscopy (FTIR) and UV–Vis spectroscopy. Color strength (K/S) and colorimetric data of the RSE dyed fabrics along with their fastness properties against washing and light were measured. Chemical, physical and thermal discrepancy between undyed and RSE dyed fabrics was monitored using FTIR, moisture regains, wettability, X-ray diffraction pattern (XRD) and Thermogravimetric analysis (TGA) along with diffraction scanning calorimetry (DSC). The alterations in the morphological structure of the plasma pretreated fabrics were studied using scanning electron microscopy. The findings of this work introduce an eco-friendly and economically feasible route for the valorization of rice straw waste as sustainable alternative to certain chemical finishing agents. RSE developed multifunctional protective textiles with effective coloration with excellent UV protection and antioxidant activity along with improved thermal stability.

## Introduction

Rice straw, a byproduct of rice cultivation, recent research efforts have been focused on recycling rice straw to develop valuable products using environmentally friendly and economically feasible processes^1–4^. These include the production of nanohybrids, antioxidants and silica/lignin- based materials which are utilized in paper production, soil improvement, paint, Rubber industry^5–9^. The antioxidant potential of rice straw extract is strongly associated with the composition of phenolic compounds^10–12^. Previous studies identified that p-hydroxybenzoic acid, vanillic acid, syringic acid, cis-p-coumaric acid, ferulic acid, sinapic acid, and related derivatives^13–17^, these phenolic compounds are sustainable, non-toxic, biodegradable, safe for human use and exhibit antimicrobial and UV-shielding properties^12,18^.

Among the textile materials, wool and silk are two widely used natural protein-based fibers because of their hygroscopicity, softness, and good mechanical properties^19,20^. On the other side, Nylon 6 (Polyamide 6) is a synthetic fiber extensively employed for its strength, elasticity, abrasion, and deformation resistance^21^.

Conventional textile dyeing is relying heavily on synthetic dyes which, despite their color brilliance and fastness, significantly contribute to environmental pollution due to their toxicity, poor biodegradability^22,23^, and resistant to wastewater treatment, these Concerns have driven increasing interest in green methods and novel natural and functional properties of wool and silk fabrics for sustainable textile coloration and finishing^24–26^.

Natural dyes are rich in phenolic, flavonoid, and tannin compounds, which not only impart coloration but also offer multifunctional properties such as UV protection, antimicrobial activity, and antioxidant properties^27–30^, making them suitable for medical, hygienic, decorative applications these functional fabrics can protect skin from aging and oxidative damage while enhancing fabric durability^31–34^. Accordingly, the incorporation of natural dyes into textile processing aligns with global trends toward sustainable production and circular economy models^11^. Moreover, previous studies have reported the use of various plant parts extracts for on-pot coloration and fishing of nylon 6 surface with UV protection, antibacterial, and flame retardant^35–39^.

In the textile industry, plasma technology is utilized as effective and green tool. It includes changing the surface properties of textile materials without changing the bulk properties by using ionized gas that contains energetic species like electrons, ions, and radicals^40^. Plasma treatments can enhance hydrophilicity, dye uptake, printability, adhesion, and even antimicrobial characteristics. The mechanism involves the generation of active sites and the introduction of polar functional groups to improve surface reactivity and compatibility with successive finishing agents or coatings^41^. Low-pressure and atmospheric pressure plasma systems have been applied with great success to a wide range of fibers, from wool, polyester, polypropylene and nylon. Consequently, these processes are becoming significant in green textile manufacturing because they are considered a cleaner and more efficient alternative for surface functionalization or finishing. Recent studies have reported the development of multifunctional textiles with combined protective and functional properties using advanced materials and processing techniques^42,43^. However, most of these approaches rely on synthetic chemicals or complex fabrication routes, while the utilization of agricultural waste derived natural dyes for simultaneous coloration and multifunctional fishing of both natural and synthetic fibers remains limited.

Based on the background, this study explores rice straw extract as a green, multifunctional natural dye for simultaneous coloration and functional fishing of wool and silk, nylon 6 fabrics. The effect of atmospheric-plasma pretreatment on enhancing fabric affinity toward the extract along with the imparted UV-protective, antioxidant, and thermal stability properties, is systematically investigated. This approach offers potential economic advantages, including low production costs and the value of sustainable textile products in emerging eco-friendly markets.

## Materials and methods

### Materials

Samples of rice straw, about 1.0 m long, were gathered from the El-Sharkia Government in Egypt. For later usage, the rice straw was cut to a size of 2.0 mm and kept at room temperature, away from heat and moisture.

Plain weaved crossbred wool fabric 140 g/m^2^, plain weaved structure nylon 6 fabric 180 g/m^2^, degummed woven natural silk fabric from Chinese silkworm Bombyx mori 85 g/m^2^ were purchased from Misr Company for Spinning and Weaving, El-Mehalla El-Kobra, Egypt.

Various chemicals and solvents have been used: Sodium hydroxide (purity: 99.0%, MW:40gm/mol, CAS:1310-73-2), ethanol (MW: 46.07, purity: 99.99%, CAS:64 -17-5 ), methanol (purity: 100%, MW:32.04, CAS:67-56-1, these were analytical-grade products that were bought from Piochem, Hydrochloric acid (CAS 7647-01-0, 37%, ACS reagent, Sigma-Aldrich) and n-butanol (CAS 71-36-3, ≥ 99%, ACS reagent, Sigma-Aldrich) were used as received.

### Methodology

#### The alkaline solar pulping of rice straw

The alkaline solar pulping of rice straw yields the black liquor used in this study it was carried out in closed polyethylene bags following the procedure. Sodium hydroxide (NaOH) was used as the pulping chemical at a concentration of 20% (w/w) based on the dry weight of the straw, with a liquor-to-fiber ratio of 10:1. The sealed bags were exposed to direct sunlight for 4 days as a natural heating source until the fibers were fully cooked. After the solar treatment period, the resulting black liquor was collected for further use, while the pulped fibers were thoroughly washed with water until neutral pH was achieved and then air-dried at room temperature^44,45^.

#### Extract from black liquor (BL)

One liter of alkaline BL solutions (pH11) was acidified to pH2 using 20% hydrochloric acid, the produced precipitate was filtered off, and the filtrate was extracted by n-butanol. The separated organic layer was washed several times with distilled water and then evaporated under vacuum to obtain the crude extract residue; the residue was further extracted with methanol. Evaporation of methanol under reduced pressure yielded 5 g of the RSE extract.

#### Fabric preparation

Wool, silk and Nylon 6 fabrics were scoured to remove any lubricating oils used during the weaving from its surface, a scouring process was carried out by immersing the fabric in an aqueous solution containing Na_2_CO_3_ (2 g/L) and nonionic detergent (1 g/L), for 15 min at 60 °C^46^. The scoured fabric was then removed and rinsed thoroughly with running water and finally dried at room temperature.

#### Plasma treatment

Wool, silk and nylon 6 fabrics were exposed to an AC air-plasma discharge unit (PPODC-001-HP; 115 V/PDC-002-HP; 230 V), coupled with a PDC-VCG (115 V)/PDC-VCG-2(230 V) vacuum gauge and digital meter. The input voltage was adjusted to 200 V at 50–60 Hz. The exposure time was 15 min at 0.8 Torr with a radio frequency (RF) power of 45 W, and the air gas flow rate was 72.3 mL/min.

#### Fabrics dyeing

Wool, silk and nylon 6 fabrics were dyed by rice straw extract using different concentrations (0.2- 0.4- 0.6–0.8%, w/v), at different pH values (4–7–9), at different temperatures (65–75–85 ℃) for 1 h with liquor ratio was 1:50 and the dyed samples were then rinsed with running water, followed by air-drying at room temperature.

### Analyses

#### The LC/MS/MS spectra of rice straw extract (RSE)

The phenolic composition of RSE was analyzed using an ExionLC AC system coupled with a SCIEX Triple Quad 5500 + mass spectrometer with electrospray ionization (ESI). Both negative ionization and MRM modes were applied for compound identification and quantification using standard C18 columns. The mobile phases and gradient programs were applied as described in previous studies^47,48^. MS/MS parameters (curtain gas, collision energy, ion spray voltage, and source temperature) were set according to instrument specifications. The injection volume was 5 µL, and the flow rate was 0.3 mL/min.

#### Characterization of the wavelength the rice straw extract (RSE)

UV–Vis spectroscopy was used to examine the extract’s absorption pattern in the UV–Visible region using JENWAY-6405 UV/V spectrophotometer (Bibby Scientific Ltd., UK) and the wavelength used was from 250 to 500 nm.

#### Color measurements

The color intensity (K/S) values of the Dyed wool, silk and nylon 6 fabrics with the rice straw extract (RSE) evaluated using a spectrophotometer with pulsed xenon lamps light source (Ultra Scan Pro, Hunter Lab, USA) 10◦ observer with D65 illuminant, d/2 viewing geometry and measurement area of 2 mm at λ_max_ 300.

#### Colorfastness to washing

The colorfastness to washing was determined according to method ISO 105-C06 (2010)^46^ using Lunder ometer. The samples (5 × 10 cm) were sewed between two similar pieces of bleached cotton fabric. The specimen was immersed into an aqueous solution containing 5 g/l non-ionic detergent and 2 g/l of sodium carbonate at a liquor ratio 1:50, the bath was thermostatically adjusted to 45 °C. The test was run for 30 min at 42 r.p.m. The samples were then removed, rinsed twice with occasional stirring or hand squeezing, then dried. The washing fastness was assessed using Grey scale reference for colour change.

#### Colorfastness to light

Colorfastness to light was determined according to AATCC test method (16 A – 1989). The evaluation was established using the blue scale as reference of colour change.

#### Fourier transform infrared spectroscopy (FTIR)

The chemical characteristics and functional groups of the examined rice straw extract (RSE) and Dyed fabrics were characterized qualitatively. The FTIR spectra were obtained using a JASCO FTIR-6000 E, (Japan). phenolic was mixed with KBr (potassium bromide) to make discs, measured in the wave number range of 4000–400 cm^**− **1^, using a resolution of 4 cm^**− **1^, and using Model ATR PRO450-S, Single Reflection Measuring Attachment for measuring fabrics.

#### Scanning electron microscope (SEM)

The morphological structure of fabrics before and after plasma-treatment was assessed using a ZEISS LEO 1530 Gemini Optics Lens SEM of 30 KV scanning voltages.

#### Ultraviolet protection factor

The ultraviolet protection factor (UPF) was automatically calculated for the Dyed as well as unDyed samples, according to Australia/New Zealand standard AS/NZS-4399:1996 method employing UPF calculation system of UV/Vis spectrophotometer as reported in the standard AATCC Test Method 183:2010-UVA Transmittance.

#### Moisture regains

Measurements of moisture regain of the fabrics were performed using the standard ASTM method 2654-76 (West, 1981). Moisture regain of the samples was calculated according to the following Eq. 1:

1 $$Moisture\; regain\; {\%} = \frac{W1-W2}{W2} \times100$$

where W1 is the weight of sample (g) after saturation in the standard humidity atmosphere; W2 is the constant weight (g) of dry sample.

#### Wettability

The fabric wettability was assessed according to the AATCC standard test method 79-2018. A drop of water was allowed to fall on the tested sample, and the time required for the drop to disappear was recorded (AATCC standard test method 79-2018).

#### Antioxidant properties

The antioxidant activity of the (RSE) and the dyed as well as the undyed samples were evaluated using the DPPH free radical scavenging assay as in^49^, with slight modification. Briefly, a 100 µl sample (concentration 50, 100, 150, 200, and 600 µg pigment/ml deionized water) and 2.5 cm^2^ of Dyed samples (wool, silk and nylon 6) were mixed with 900 µl of 0.1 mM DPPH solution in methanol and incubated in the dark for 30 min at 37 °C. After incubation time, DPPH decolorization was determined by measuring the absorbance at λ_max_ 517 nm. DPPH radical scavenging activity was calculated using the given Eq. 2.

2 $$Inhibition\; (\%) =\;\frac{{A}_{1}-{A}_{2}}{{A}_{1}}\times100$$

where A_1_ was the absorbance of the DPPH solution without the sample and A_2_ was the absorbance of DPPH with the sample.

#### X-ray diffraction pattern (XRD)

XRD data were measured by the modern diffractometer Bruker d8 advance, Germany, using copper source Kα radiation (λ = 1.5406 Å) at 40 mA, 40 kV, in the 2θ range 5°–80°, step size 0.05° using automatic divergence slit and scan rate of 0.6°/sec.

The crystallinity Index (CI %) index was calculated by the XRD peak-deconvolution integration (area) method, according to Eq. (3). The diffractograms were deconvoluted into crystalline and amorphous components using Origin Pro, and peak areas were obtained after background subtraction and curve fitting (Gaussian functions).

3 $$CI\left(\%\right)=\left\{{A}_{cr}/\left({A}_{cr}+{A}_{am}\right)\right\}\times 100$$

where A_cr_ is the summed area of fitted crystalline peaks and A_am_ is the area the summed area of the amorphous halo in the diffractogram^50^.

#### Thermal properties

Thermogravimetric analysis (TGA) was measured using SDT Q2000 Tzero and diffraction scanning calorimetry (DSC) from TA instruments under nitrogen atmosphere with a rate of heating 10 °C/min.

#### Durability test

The durability of dyed fabrics was evaluated in terms of UPF and antioxidant properties of the samples. Adopting the standard AATCC 61-1989 method, the washing durability of the finished fabrics was examined. The Dyed fabrics were washed for 1, 5, 10, and 20 wash cycles. The Dyed fabric (5 × 15 cm) was mounted in a launder-o-meter with a detergent solution (200 mL) at 40º C for 45 min.

## Results and discussion

### The full-scan LC/MS/MS spectra of rice straw extract (RSE)

The chemical profiling of the rice straw extract (RSE) was initially performed using a full-scan LC/MS/MS analysis to detect the broadest possible range of phenolic and flavonoid compounds in the extract. Qualitative screening enables the detection of all ionizable compounds with the selected mass range, giving a complete fingerprint of phytochemical constituents. The identification of the compounds (Table 1) was based in the negative [M-H]—mode and the product ion [Ms-Ms ions] which align with previously identified compounds in literatures^12^.

The full scan detected a total of sixteen compounds, primarily belonging to phenolic acid (e.g., p-Coumaric acid, Vanillic acid, Ferulic acid, trans-3-Hydroxycinnamic acid, Caffeic acid, Syringic acid, Vanillin, Chlorogenic acid, 3,4-Dihydroxybenzoic acid, Sinapinic acid, and Protocatechuic acid, while flavonoids detected such as naringenin, hesperetin, Catechin, kaempferol, and methyl gallate^12,18^. These compounds are commonly associated with the lignocellulosic structure of rice straw and originate from partial depolymerization of lignin and hydroxycinnamic acid derivatives.

**Table 1:** Major phenolic compound and flavonoids in Rice straw extract (Full-scan LC/MS/MS).

Phenolic compounds	Rt (min)	Molecular formula	[M-H]^–^(m/z)	Main fragment ions(m/z)	Database code
p-Coumaric acid	10.18	C_9_H_8_O_3_	163.0	119.04 163.0	MSBNK-RIKEN-PR309364
Ferulic acid	6.871	C_10_H_10_O_4_	193.0	178.0 149.0 134.0 117.0 107.0 106.0	MSBNK-RIKEN-PR309325
Caffeic acid	5.359	C_9_H_8_O_4_	179.0	179.0 135.02 134.06	MSBNK-RIKEN-PR100533
Syringic acid	2.484	C_9_H_10_O_5_	197.0	197.0 181.0 167.0 137.0 121.0 123.0 105.9	MSBNK-Keio_Univ-KO001813
Trans-3-Hydroxycinnamic acid	10.180	C_9_H_8_O_3_	163.0	163.0 119.0	MSBNK-RIKEN-PR305613
Vanillin	3.166	C_8_H_8_O_6_	151.0	151.0 136.0 124.0 109.0 108.0 107.0	MSBNK-MetaboLights-ML005951
3,4-Dihydroxybenzoic acid	3.109	C_7_H_6_O_4_	153.0	108.0 109.0 153.0	MSBNK-RIKEN_ReSpect-PT203280
Vanillic acid	2.103	C_8_H_8_O_4_	167.0	108.0 124.0 152.0	MSBNK-BS-BS003124
Protocatechuic acid	3.109	C_7_H_6_O_4_	153.0	153.0 109.0	MSBNK-RIKEN_ReSpect-PM000402
Sinapinic acid	5.263	C_11_H_12_O_5_	223.0	223.0 195.0 193.0 197.0 163.0 164.0 135.0	MSBNK-BGC_Munich-RP017412
Catechin	6.444	C_15_H_14_O_6_	289.0	215.0 151.0 123.0 107.0	MSBNK-BS-BS003014
Kaempferol	11.659	C_15_H_10_O_6_	285.0	285.0 269.0 210.0 211.0 121.0	MSBNK-LCSB-LU089855
Naringenin	8.463	C_15_H_12_O_5_	273.0	201.0 187.0	MSBNK-Washington_State_Univ-BML00692
Hesperetin	8.273	C_16_H_14_O_6_	3030	287.9 232.0 188.07	MSBNK-RIKEN-PR309441
Methyl gallate	10.05	C_8_H_8_O_5_	184.1	124.0	PM012531

### Quantified phenolic compounds and flavonoids in rice straw extract (RSE)

Following the qualitative screening, a targeted quantitative analysis was conducted using Multiple Reaction Monitoring (MRM) mode. For this purpose, twenty authentic standards were analyzed under the same conditions, and the extracted sample was subsequently evaluated to determine which of these compounds were present at quantifiable levels. The targeted LC/MS/MS analysis in MRM mode enabled the accurate quantification of ten phenolic compounds detected in the extract (Table 2). The results showed that ferulic acid and Syringic acid were dominating phenolics with concentrations of 9484 µg/mL and 3432 µg/mL, respectively. This agrees with previous reports that ferulate and syringate derivatives are major structural lignin-bound phenolics in cereal residue. Moderate concentrations were observed for p-coumaric acid (667.6 µg/mL), caffeic acid (148.3 µg/mL), vanillin (187.8 µg/mL), Naringenin (186.2 µg/mL), and 3,4-Dihydroxybenzoic acid (153.5 µg/mL), reflecting their partial release during extraction.

In contrast flavonoids such as hesperetin, kaempferol, and methyl gallate appeared at very low levels components with their minor natural abundance in rice straw compared to hydroxycinnamic and hydroxybenzoic acids. The noticed retention times closely coincided with the expected values, confirming reliable chromatographic separation and correct identification. Overall, the results evidence that rice straw extract (RSE) is mainly rich in hydroxycinnamic acids, particularly ferulic and Syringic acid that could contribute very importantly to their antioxidant and functional properties.

These phenolic compounds may be utilized in textile applications because they exhibit strong antioxidant activities, which contribute to improved fabric durability. Additionally, flavonoids such as kaempferol and naringenin are natural UV absorbers, improving the UV- protective performance of Dyed textiles.

The interaction of these compounds with fibers can also allow hydrogen bonding and π-π staking, which may increase fiber crystallinity, rigidity, and hydrophobicity, improving the overall physical stability of the fabric^51–53^. Therefore, the phenolic and flavonoid profile of the rice straw extract (RSE) demonstrates its potential as an effective bio-fishing agent for imparting multifunctional properties to textile materials.

**Table 2:** Major phenolic and flavonoid compounds in rice straw extract (RSE) using targeted quantitative analysis.

Compounds	Expected RT(min)	RT(min)	Concentration (µg/mL)
Caffeic acid 178/135	5.83	6.14	148.3
Ferulic acid 192.8/133.9	9.29	9.29	9484
Coumaric acid 162.9/119	7.98	7.98	667.6
Vanillin 151/136	7.84	7.84	187.8
Naringenin 271/151	20.98	21.4	186.2
3.4-Dihydroxybenzoic acid 152.9/109	3.13	3.38	153.5
Hesperetin 301/136	22.62	22.52	0.6951
Methyl gallate 183/124	5.33	5.33	0.3031
Kaempferol 284.7/93	22.4	22.4	4.694
Syringic acid 196.8/181.9	6.54	6.54	3432

### UV–visible characterization of the rice straw extract (RSE)

**Fig. 1:**
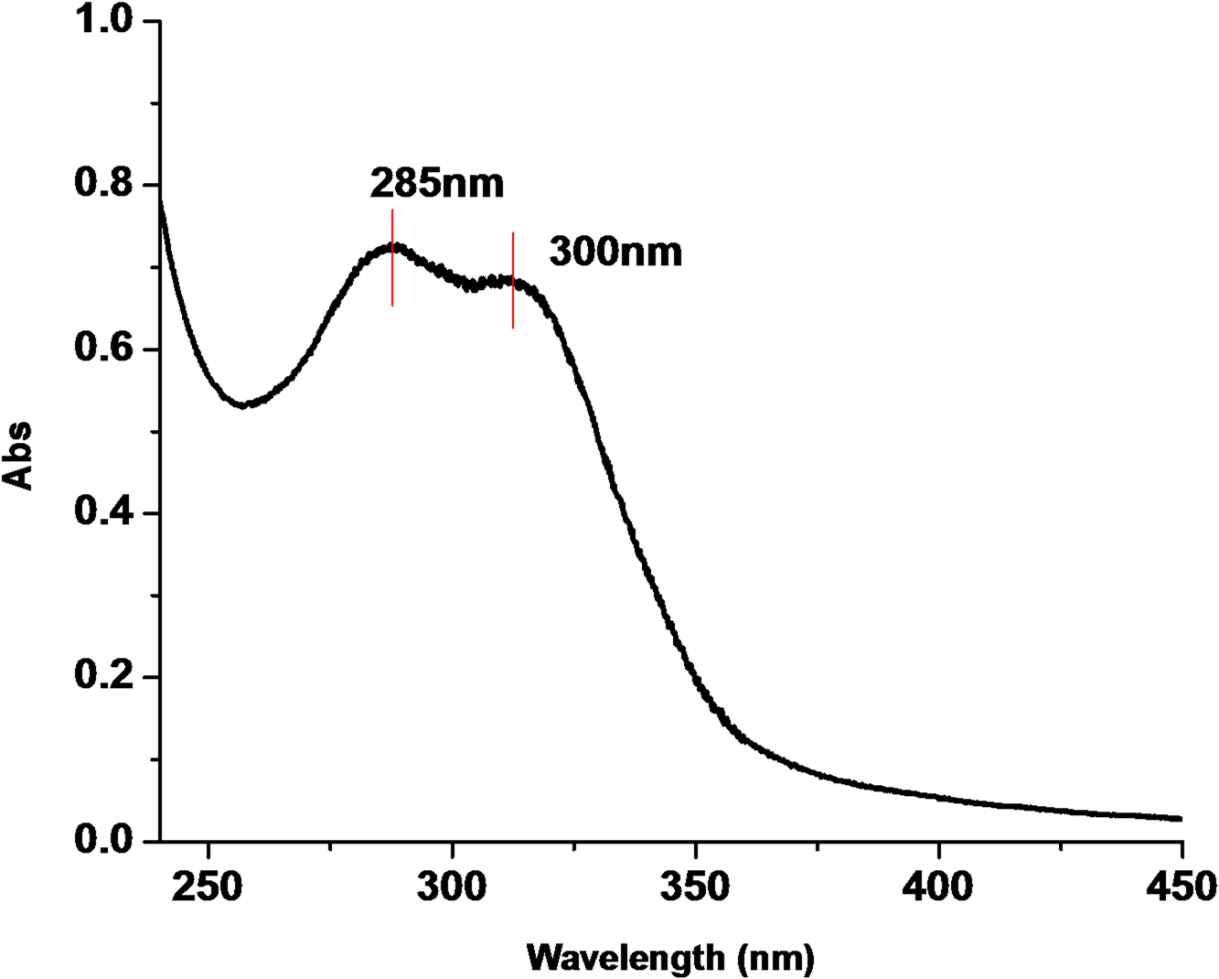
UV-Visible spectrum of rice straw extracts (RSE).

Figure 1 shows the UV-Visible spectrum of rice straw extract (RSE). The figure indicated the wavelength of maximum absorption (λ max) at two distinct wavelengths, one in UV region at 285 nm and other in at 300 nm. The two absorption wavelengths were correlated to π-π^*^ and n- π^*^ transitions respectively. The π-π^*^ transition state occurs in molecules with conjugated system; it requires high energy and appears at shorter wavelength (i.e. the conjugated systems of the benzene ring of the phenolic compounds). While n- π^*^ transitions occur in molecules with lone pair of electrons and π bonds; it requires less energy and appears at longer wavelengths (i.e. lone pairs of oxygen on the phenolic compound).

### Color measurements

Wool and silk as natural fibers are composed of amino acids and nylon 6 as well although it is a synthetic fiber it composed of a repeating amide linkage with terminal amino and carboxylic group at each end of the polymeric chain.

**Fig. 2:**
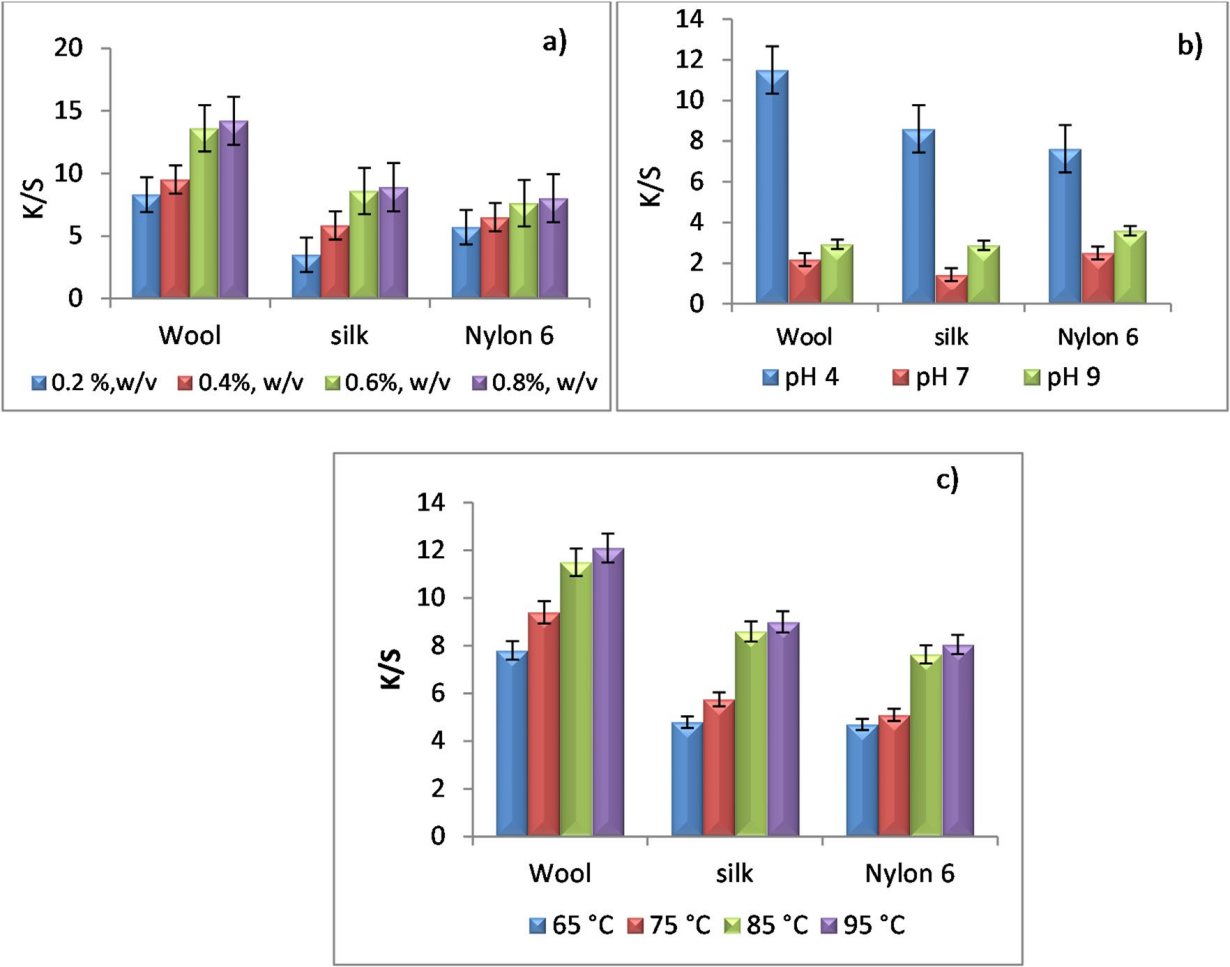
The color strength values under different reaction conditions, (**a**) different concentration, (**b**) different pH values, and (**c**) different temperatures.

Based on the chemistry of the examined fabrics, the effect of different dyeing conditions was studied and was shown in Fig. 2.

**Fig. 3:**
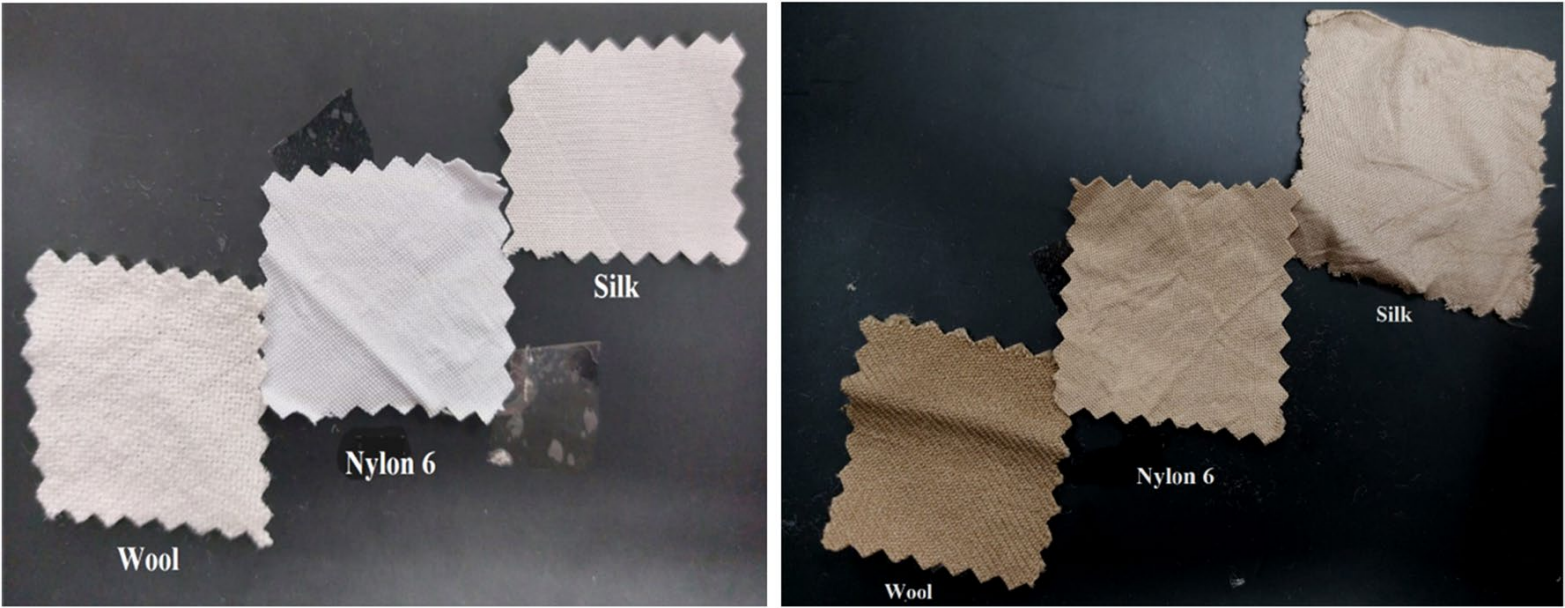
Photographs of the blank and dyed fabrics with RSE on the right and left side respectively.

**Scheme 1:**
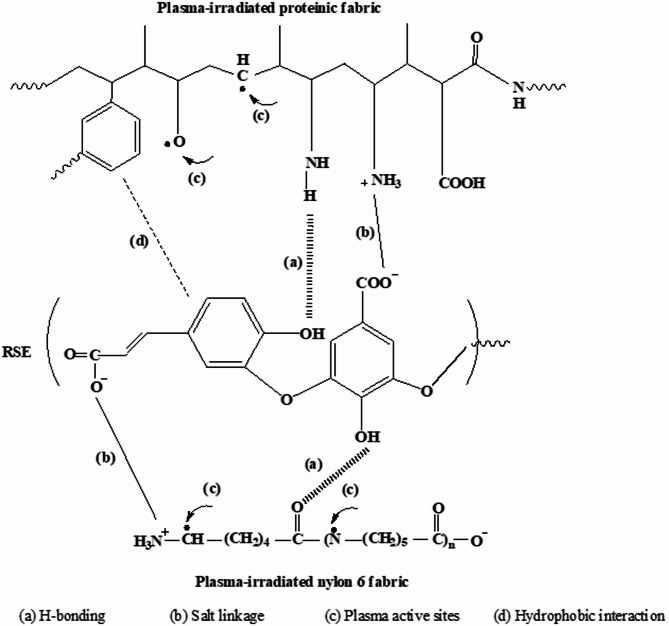
Schematic diagram of the suggested interactions between plasma-treated fibers and phenolic compounds.

Figure 2-a showed that the increase in the concentration of the extract leads to increasing in its absorption and hence a higher color strength value. Additionally, the affinity of the extract to dye wool, silk and nylon 6 fabrics is higher at pH 4 as observed Fig. 2-b, which implies that the rice straw extract (RSE) contains functional groups that are negatively charged. This may be explained due to the ionic bonding interaction (salt linkage) between the negatively charged dye molecules and the positively charged protonated-amino groups in the fabric in acid medium (cf. Scheme 1). The effect of treatment temperature was also studied and presented in Fig. 2-c that revealed that the color strength values increased by increasing the temperature. Based on the aforementioned results it can be concluded that the optimum conditions of the dyeing of wool, silk and nylon 6 fabrics with the rice straw extract (RSE) are 0.6%, w/v concentration, at pH 4, with L.R 1:50, at 85 °C, for 1 h and photos of the blank and dyed fabrics can be seen in Fig. 3.

In another attempt the effect of the pretreatment of the fabrics by exposure to air plasma followed by dyeing by RSE and the optimum conditions were attained.

Results of this study were presented in Table 3. Table 3 shows a significant increase in color strength compared to the dyed ones. Table also revealed that plasma-pretreated fabrics have small lightness (L*) values which are correlated to the high color strength values compared to those without plasma pretreatment. Furthermore, the color depth is more shifted to yellow area and less to red area as declared by the values of a* and b*, and a high apparent depth for plasma pretreated samples.

The superior color strength data of the air plasma-pretreated fabrics exposure could be rationalized by the activation of the fabric surface and introducing new functional groups (viz., –OH and –COOH) which introduce a new chemical bond between the fabrics and the molecules of rice straw extract (RSE) [45] (cf. Scheme 1). Besides air plasma exposure removes the lipid layer and contamination on fabric making the surface more porous and hydrophilic which facilitates the diffusion of the extract more inside the fabrics^54^.

**Table 3:** Color coordinate values (L*, a*, b*) of dyed fabrics with and without plasma pretreatment.

Sample	K/S	L*	a*	b*
Wool, pH 4, 0.6%, 85 °C, 1 h (Dyed1*)	11.5	47.95	5.16	18.18
Silk, pH 4, 0.6%, 85 °C, 1 h (Dyed1*)	8.6	62.31	3.84	15.49
Nylon 6, pH 4, 0.6%, 85 °C, 1 h (Dyed1*)	6.6	60.92	5.27	18.87
Wool, pH 4, 0.6%, 85 °C, 1 h (Dyed2*)	17.1	43.47	7.08	21.94
Silk, pH 4, 0.6%, 85 °C, 1 h (Dyed2*)	13.8	55.77	6.74	22.99
Nylon 6, pH 4, 0.6%, 85 °C, 1 h (Dyed2*)	10.7	43.02	10.45	24.49

Dyed 1*: is the dyed fabric with the extract without plasma pretreatment.

Dyed 2*: is the dyed fabric with the extract with plasma pretreatment.

### Colorfastness properties

Data of Table 4 shows the washing and light fastness of the dyed and plasma-pretreated samples. The table shows that the samples dyed after exposure to air plasma have better fastness properties than those without plasma exposure which emphasize the strong bonding between the fabrics and the rice straw-extract either by salt linkage or H-bonding. It is also worth mentioning that light fastness properties of the samples dyed after exposure to plasma improved, as the light fastness is increased with increasing the depth of color as it is correlated to the functional group responsible for the coloration. Based on the light fastness data it was recommended to use the dyed fabrics as indoor clothes and avoid exposure to sunlight.

**Table 4:** Fastness properties of wool, silk and nylon 6 fabrics dyed with the extract with and without air plasma exposure.

Sample	Washing			Light fastness
	Staining		Alt.	
	St*	St**		
Wool-Dyed 1*	4	4	4	4
Silk-Dyed 1*	4	4	3–4	4
Nylon 6-Dyed 1*	4	4	3–4	4–5
Wool-Dyed 2*	4–5	4–5	4–5	4–5
Silk-Dyed 2*	4–5	4	4	4–5
Nylon 6-Dyed 2*	4	4	4	5

st*, staining on cotton; st**, staining on wool; Alt., Alteration in color; Dyed 1*, is dyed fabric with the extract without plasma pretreatment; Dyed 2*, is dyed fabric with the extract with plasma pretreatment.

### Scanning electron microscope (SEM)

**Fig. 4:**
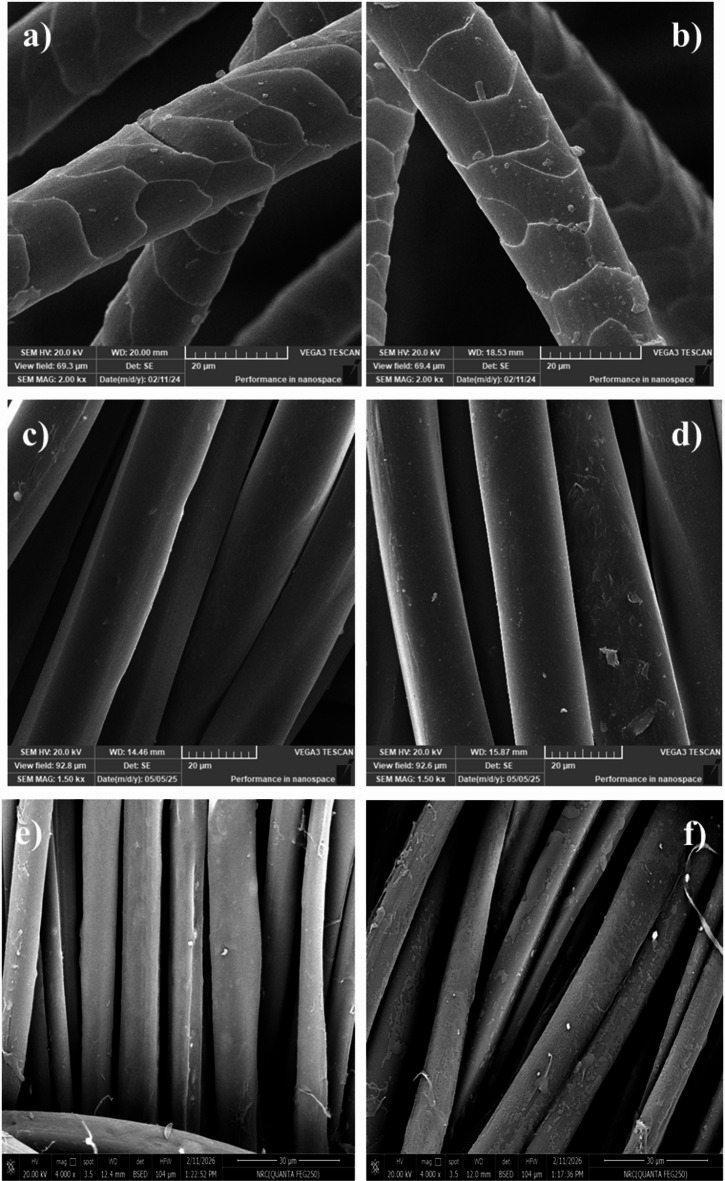
The scanning electron micrographs of blank and (**a**) blank, (**b**) plasma-treated wool, (**c**) blank, (**d**) plasma-treated nylon 6, and (**e**) blank, (**f**) plasma-treated silk fabrics.

Surface morphology of the blank and plasma-treated fabrics was assigned via SEM images in Fig. 4. The SEM images showed cracks in the scales of wool fiber and in the surface of silk and nylon 6 fabrics as well. Plasma pre-treatment of the wool fabrics activated fiber surface by the removal of the hydrophobic lipid layer as well as oxidation of cystine moieties in wool, forming hydrophilic groups. The pretreatment was shown on the surface of nylon 6 and silk fabrics also as small cracks and roughness on the surface of fabrics.

### Fourier transform infrared spectroscopy (FTIR)

**Fig. 5:**
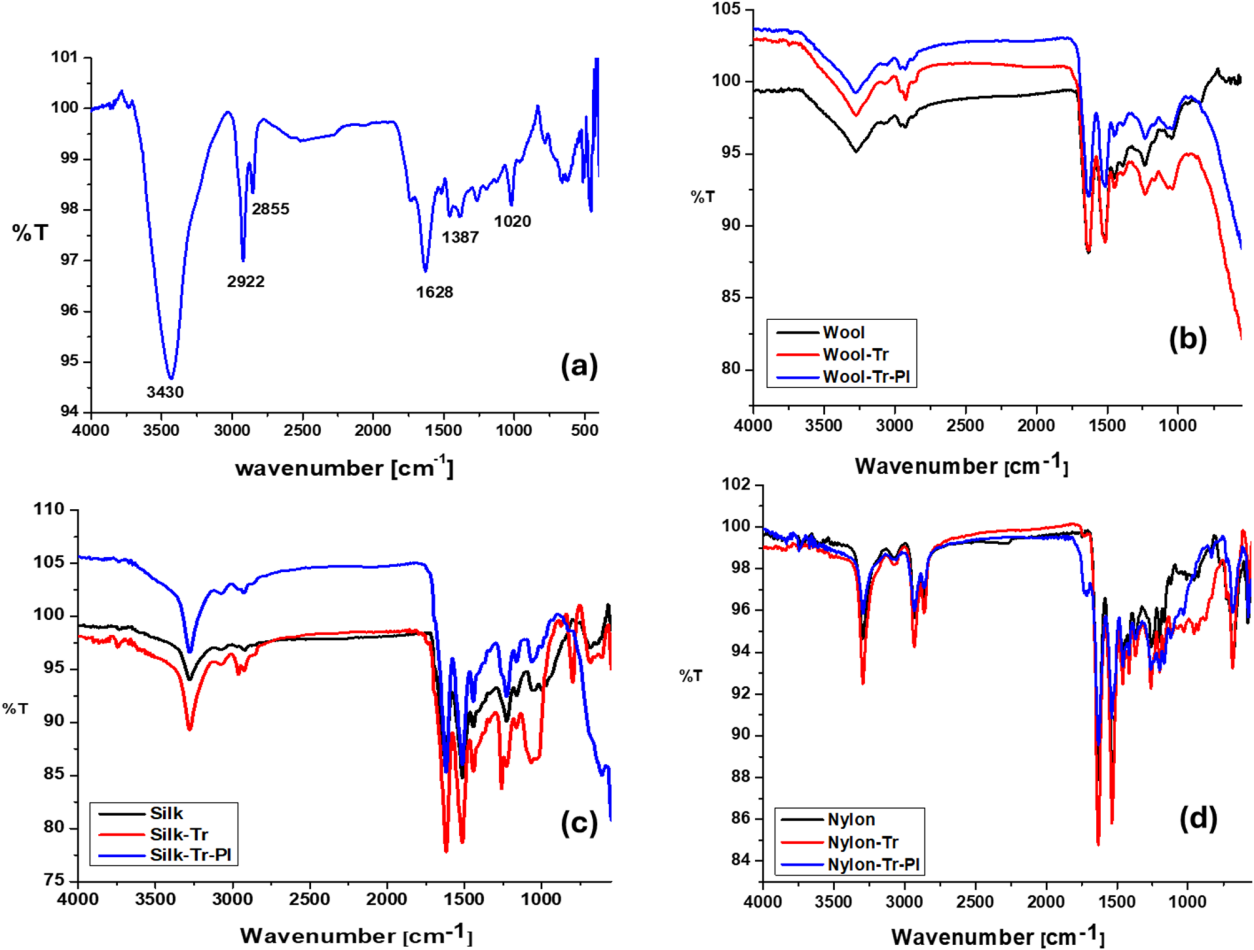
The FTIR of (**a**) the extract of Rice straw, (**b**) dyed and undyed wool fabric, (**c**) dyed and undyed silk fabric, (**d**) dyed and undyed nylon fabric.

The characteristics band of rice straw extract (RSE) is shown in the FT-IR spectra Fig. 5-a. A broadband detected at 3430 cm^− 1^[9], which was attributed to stretching vibrations of the alcoholic and phenolic OH groups of phenolic compounds. Bands of 2922 cm^− 1^ indicated the presence of alkyl groups. Also 1731 and 1628 cm^− 1^ were attributed to the C=O and C=C groups of phenolics^9^. Band at 1020 cm^−1^indicate presence of bending vibration of C–H, while 1196 cm^−1^for bending vibration of the C-OH.

Figure 5(b-d) clarified that there is no change in the primary structure of the fabrics upon using the rice straw extract (RSE). The usual bands of wool at 3276, 1631, 1515, 1235 cm^− 1^ and that of silk at 3278, 1621, 1511, 1226 cm^− 1^ which corresponds to O–H stretching, amide I connected to C=O stretching vibration, amide II of N–H bending vibration and amide III band due to the C−N stretching and N−H in-plane bending vibrations^55^. Additionally, the main characteristic bands of nylon 6 are at 1633 cm^− 1^ which is related to the carbonyl group stretching vibration of amide I, 3295 and 1535 cm^− 1^ assigned to N–H stretching and bending vibration band of amide II, and 1257 cm^− 1^ which corresponds to C–N stretching band. The figure showed that all these bands remained unchanged after the dyeing process^56^.

Referring to Table 5, which exhibits the transmittance of the groups before and after dyeing, the infrared spectral behavior of the fabrics dyed with a rice straw extract (RSE) varied noticeably depending on whether they were plasma-pretreated or not. In native fabric, the surface possesses limited reactive sites and remains relatively hydrophobic; therefore, interaction with the extract occurs mainly through weak physical adsorption or loose hydrogen bonding^57,58^. As a result, the functional groups of both the fabric and the phenolic compounds retain higher vibrational freedom, producing stronger IR absorption bands and consequently lower transmittance values. In the contrary, plasma pretreatment oxidizes and activates the fabric surface by introducing polar groups such as –OH, –COOH, and –C=O, which participate in stronger chemical and hydrogen bonds with the extract molecules. These new interactions restrict the vibrational motion of the functional groups and may also modify surface roughness, leading to weaker IR absorptions (higher transmittance). This finding matched with the higher color strength and fastness of the dyed fabrics results (cf. Table 3), confirming that plasma activation enhances the overall bonding strength and surface adhesion of the phenolic compounds and flavonoids.

**Table 5:** Transmittance percent of some bands in the FTIR spectra of undyed as well as dyed fabrics.

FTIR peaks at	Transmittance %		
	Blank wool	Wool-Treated1*	Wool-Treated 2*
3276 cm^− 1^	95	96.5	99.2
1631 cm^− 1^	88.5	84.6	95
1513 cm^− 1^	89	86.2	93.2
1234 cm^− 1^	95	90.2	97
	Blank silk	Silk-Dyed1*	Silk-Dyed 2*
3278 cm^− 1^	94	88	97.5
1621 cm^− 1^	86	77	95
1511 cm^− 1^	84.5	78.2	94
1226 cm^− 1^	90	85	95.2
	Blank nylon 6	Nylon-Dyed1*	Nylon-Dyed 2*
3295 cm^− 1^	95.2	90.4	97.4
1633 cm^− 1^	88.1	83.5	90.6
1535 cm^− 1^	88.3	85	91.1
1257 cm^− 1^	94.5	91.2	95.1

Dyed 1*: is the dyed fabric with the extract without plasma pretreatment.

Dyed 2*: is the dyed fabric with the extract with plasma pretreatment.

### Ultraviolet protection factor

The exposure of skin to solar UV radiation may cause severe skin disease viz., sunburns, premature skin aging, keratosis, and in some extreme cases, skin cancer. Solar UV light radiation contains three types of radiation, UV-A (400 –315 nm), UV-B (315 –290 nm), and UV-C (290 –200 nm). UV-A and UV-B are the most harmful for skin as UV-C are almost absorbed by the ozone layer. Native fabrics have poor UPF values and thus cannot offer sufficient protection for the skin against the solar radiation^59^.

Table 6 revealed significant increase in UPF values, and high decrease in UV-A and UV-B by the application of rice straw extract (RSE) that contains phenolic and flavonoids compounds. This can be attributed to the UV-absorbing ability of natural dye chromophores, including conjugated systems in phenolic and flavonoid aromatic structures^60^. Data of the table shows the dyed wool, silk and nylon 6 fabrics have excellent protection against UV radiation according to AS/NZS 4399:1996 standard values for textile shielding especially for samples pretreated with air plasma which increased the dye uptake and color depth, and consequently improved the UV-blocking efficiency of the dyed fabrics, as reflected by higher UPF values.

**Table 6:** UV protection characteristics of the undyed as well as dyed wool, silk and nylon 6 fabrics.

Sample	UPF	T(UV-A)	T(UV-B)
Blank wool	13.5	12.6	6.2
Wool-Dyed1*	895	0.1	0.1
Wool-Dyed2*	946	0.1	0.1
Blank silk	1.1	45.1	81.6
Silk-Dyed1*	7.5	22.1	36.9
Silk-Dyed2*	13.6	5.3	9.5
Blank nylon6	2.3	36.2	41.7
Nylon 6-Dyed1*	72.6	0.8	1.7
Nylon 6-Dyed2*	79.9	0.6	1.3

Dyed 1*: is the dyed fabric with the extract without plasma pretreatment.

Dyed 2*: is the dyed fabric with the extract with plasma pretreatment.

### Physical properties of the dyed fabrics using RSE

Table 7 shows the wettability (measured in seconds) and moisture regain % of the blank as well as dyed fabrics after plasma-pretreatment. The table shows that the wettability of the dyed fabrics highly decreased compared to the pristine fabrics and this can be attributed to the activation of the surface of fabrics by plasma irradiation forming new polar groups viz., –COOH, –OH, –NH_2_ causes its high wettability. Moreover that, the moisture regain % of the dyed fabrics found to be decreased in case of wool and silk fabrics as a result of the high affinity of the pretreated fabrics with the additional functional groups to chemically bonding with RSE which lead to the consumption of the most of polar groups in the reaction. While nylon 6 fabric shows increase in the moisture regain as a result of the introduction of the functional groups viz., –COOH, –OH of the phenolic and flavenoids to the surface of the fabric as the strength of reaction is definitely not strong as in case of wool and silk and that was obvious from the color data.

**Table 7:** the wettability and moisture regain of the blank and dyed fabrics.

Sample	Wetability (S)	Moisture regain (%)
Blank wool fabric	Up to 900	15.3
Dyed wool fabric*	90	10.6
Blank silk fabric	20	13.6
Dyed silk fabric*	5	12.22
Blank nylon 6 fabric	30	5.1
Dyed nylon 6 fabric*	7	10.2

*Dyeing after plasma pretreatment.

### Antioxidant activity of the extract-dyed fabric

**Fig. 6:**
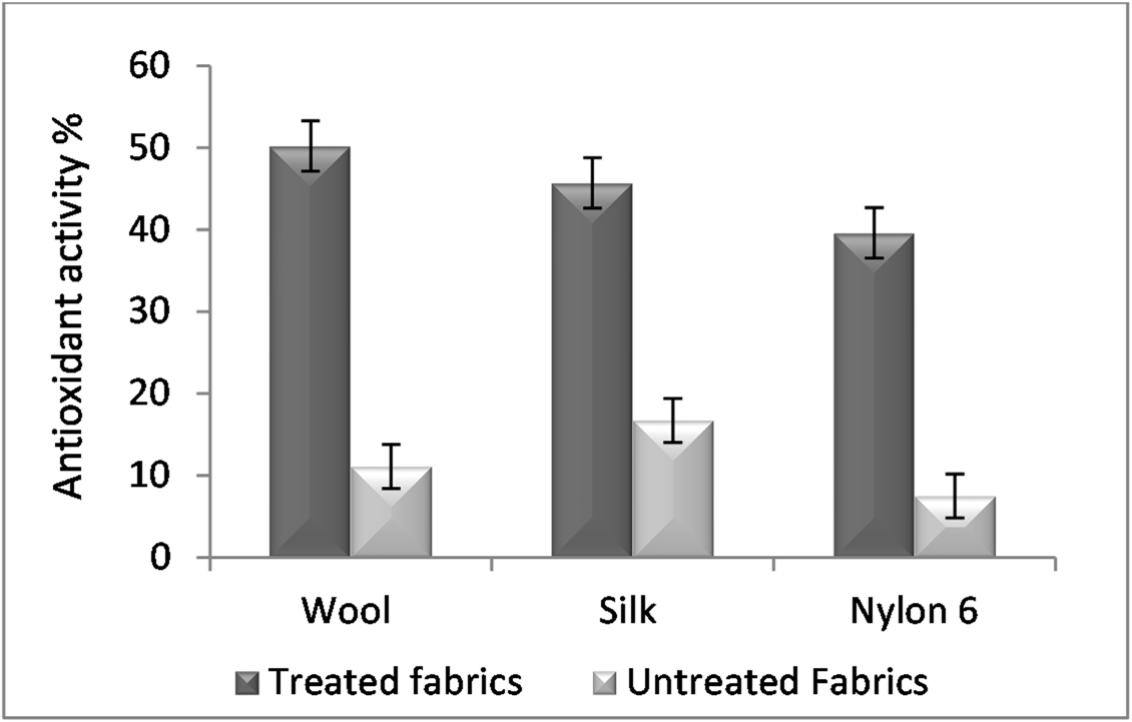
Antioxidant activity of the dyed fabrics as well as blank fabrics.

Figure 6 shows the antioxidant activity of the dyed fabrics using the rice straw extract (RSE) compared with the native ones. Although the rice straw extract (RSE) shows a weak antioxidant activity according to its IC_50_ evaluation which ranged from 100 to 200 µg/ml^61^, the antioxidant activity of the extract-dyed fabrics showed a high ability to struggle DPPH radicals compared to the native ones. This activity is most likely caused by the enhancement of the fabrics with conjugated double bond, hydroxyl, and phenolic systems present in the extract which help in the stabilization of free radical form of DPPH^62^.

### Fabric durability towards RSE

Table 8 shows the durability of dyed fabrics with RSE for UPF and antioxidant activity. It is clearly observed that the UPF value and antioxidant activity of the dyed fabrics were slightly decreased after 20 washing cycles by 9–18% and 10–15%, respectively. This indicates that RSE formed strong chemical bonds with fabric as proved in the fastness properties results in Table 4.

**Table 8:** UPF and antioxidant activity (%) of the plasma dyed fabrics after repeated washing cycles.

Sample	Washing cycle	UPF	Antioxidant activity (%)
Dyed Wool	0	946	50.2
	1	939	50.1
	5	922	49.8
	10	895	48.02
	20	855	45.1
Dyed Silk	0	13.6	45.7
	1	13.4	44.2
	5	12.8	42
	10	11.7	40.8
	20	11.1	38.9
Dyed Nylon 6	0	79.9	39.6
	1	77.8	39.1
	5	75.2	38.03
	10	73	36.2
	20	69	34.9

### X-ray diffraction pattern (XRD)

**Fig. 7:**
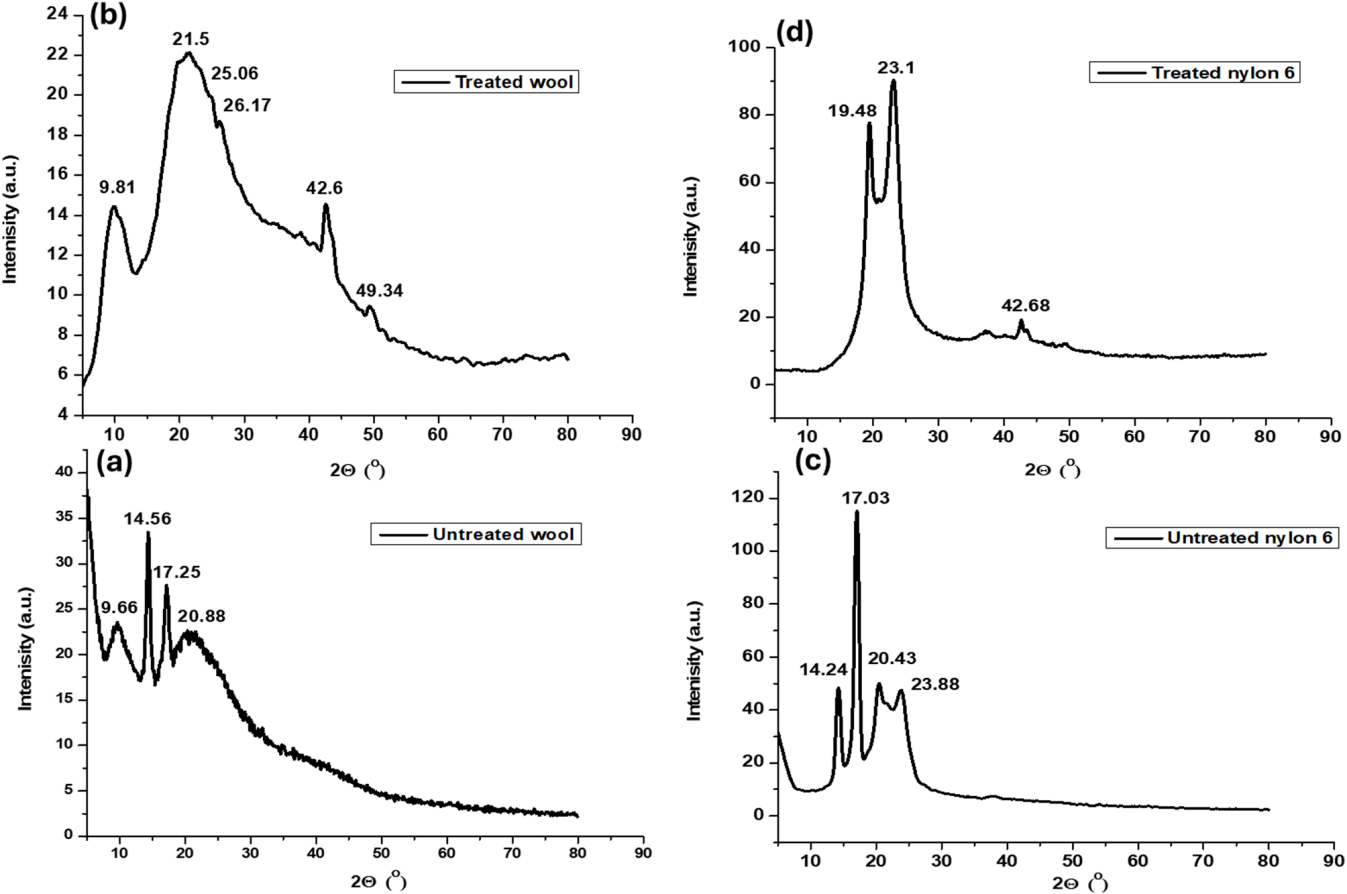
XRD spectra of (**a**) blank wool, (**b**) wool-RSE, (**c**) blank nylon6, and (**d**) nylon6-RSE fabrics.

Figure 7 shows the XRD spectra of dyed and blank wool (as an example for the proteinic fabrics) and nylon 6 fabrics. Figure 5(a-b) shows the spectrum of dyed and blank wool fabrics, the spectrum showed prominent peaks at 2θ = 9.6° and 21.5° which represents the α-helical structure with spacing of 4.4 and 9.8 A°, whereas the β-sheet structure of wool fabric shown in peaks at 2θ = 14.5° and 17.2° with the crystalline spacing 6.04 and 5.06 A°, respectively, found to be diminished after treatment with the extract. Moreover, the wider peaks implied smaller crystalline structures in the fabrics, the previous observations proved decrease in the CI % to be 40% compared to the blank wool fabric which was 55% as calculated by the Gaussian functions^63^.

The decrease in the CI% of the dyed wool fabric can be rationalized by the disruption of the disulphide bonds by the action of plasma treatment and thus disorder the crystalline regions and decrease the CI%^64^.

The diffractograms in Fig. 7c,d show the XRD spectrum of dyed and blank nylon 6 fabrics, the two characteristic peaks at 2θ = 20.4° and 23.8° assigned for the α-phase structure. On the contrary to wool fabrics, the increase in crystallinity index CI% from 50% to 58% can be attributed to strong intermolecular interactions of the phenolic and flavonoid compounds with polymeric chain of nylon 6 via H-bonding, covalent bonding or even ionic interaction after plasma pretreatment of nylon 6 fabric. That causes the formation of new cross-links between nylon6 fabrics and the extract^55^.

### Thermal properties

**Fig. 8:**
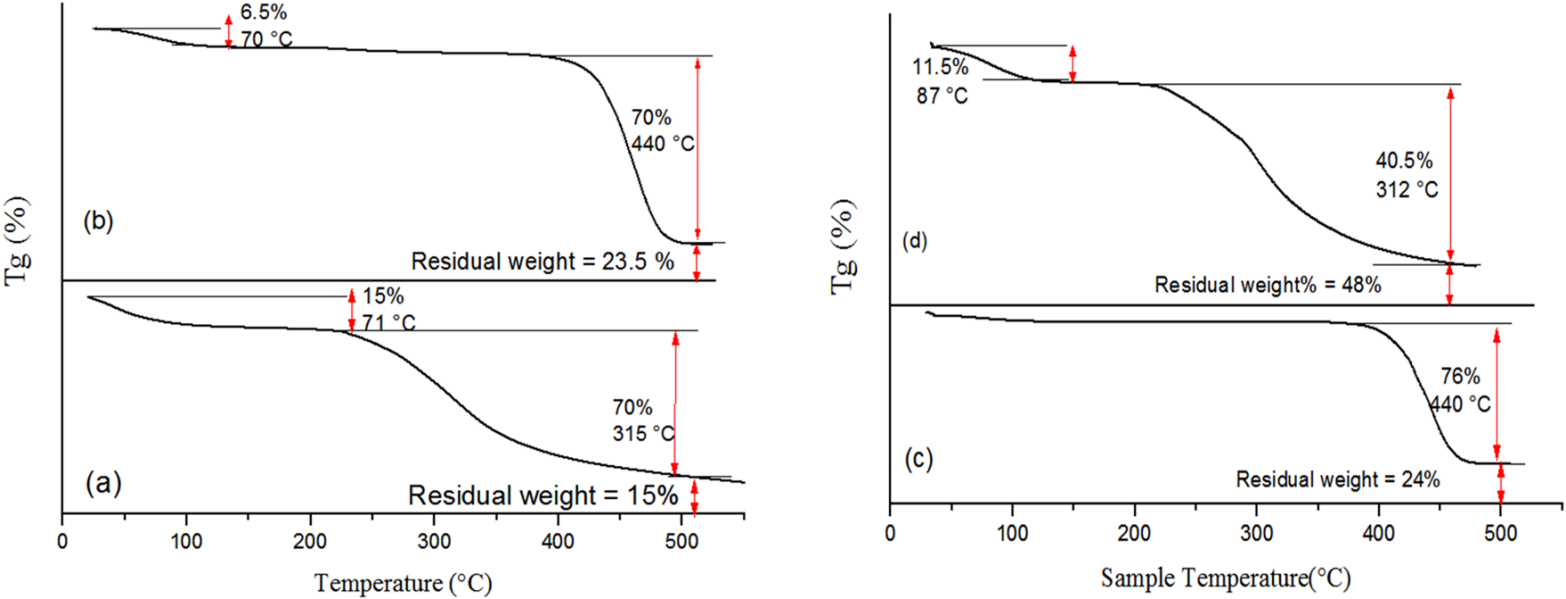
TGA charts of (**a**) blank wool, (**b**) dyed wool, (**c**) blank nylon 6 and (**d**) dyed nylon 6 fabrics.

Figure 8 shows the TGA charts of dyed as well as blank wool and nylon 6 fabrics. Figure 8a,b showed a decrease in the initial weight loss (related to moisture) in the dyed wool fabric compared to the blank one from 15 to 6.5%. That decrease in the initial loss in weight which is related to the moisture content of the fabric after dyeing proves that the fabric surface turned into less hydrophilic. This can be attributed to the consumption of the hydrophilic groups present in the macromolecular chains of wool fabric viz., –OH, –COOH and –NH_2_ in the reaction with the RSE dye via the chemical and hydrogen bonds (cf. Table 5).

Additionally, the maximum decomposition in both blank and dyed wool fabric was 70% took place at temperatures 315 ℃ and 440 ℃, with maximum rate of weight loss 55%/min and 22%/min, respectively. Finally, the dyed fabric leaves more residual char (23.5%) compared to the blank one (15%). The data in figure is a clear indication of improved thermal stability of wool fabric after treatment with rice straw extract (RSE).

Figure 8c,d represents the TGA chart of blank as well as dyed nylon 6 fabrics. Nylon 6 is a hygroscopic fabric with often undetectable moisture content ~ 2–3% through hydrogen bonding on its amide groups (cf. Figure 8c)^65^. Additionally, chart reveals maximum decomposition of ~ 76% at 440 °C and residual char ~ 24%. After the treatment of the fabric with the extract rich in phenolic and flavonoid compounds there is a significant initial loss in weight ~ 11%, this loss can be rationalized by the surface of the fabric becoming more polar and offering additional adsorption sites. Consequently, dyed fabric retains a higher moisture content compared to the blank one^66,67^ in addition to some volatile un-reacted species of the extract which could contribute ~ 11% initial loss in weight. Further investigation in the TGA chart of the dyed fabric showed an early decomposition for the sample which also could be correlated to the unbounded or un-reacted compounds from the natural extract. The thermal stability of the dyed fabric compared to the blank one arises mainly from the lower maximum decomposition (i.e. ~ 40%) in spite of the early decomposition which shows a strong control to the thermal degradation, in addition to the higher char residue which implies better thermal resistance. Finally, although the DTG maximum peak temperature was higher in the blank sample, the rate of decomposition was obviously slower in case of the dyed sample (~ 5.37%/min) compared to the untreated sample (~ 24.5%/min).

**Fig. 9:**
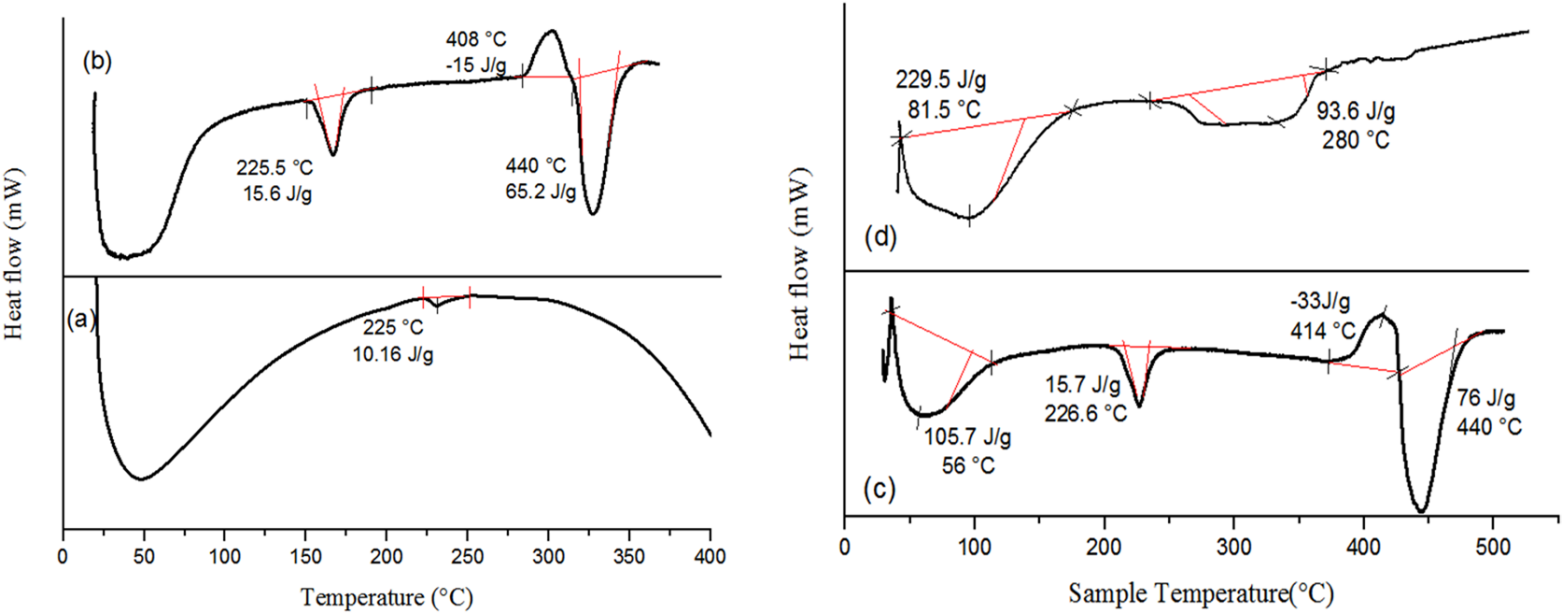
DSC charts of (**a**) blank wool, (**b**) dyed wool, (**c**) blank nylon 6 and (**d**) dyed nylon 6 fabrics.

Figure 9 represents the DSC charts of the blank as well as dyed wool and nylon 6 fabrics. The increase in the heat enthalpy ∆H of the dyed wool fabric at 225 ℃ from ~ 10 J/g to ~ 15.5 J/g proved that the fabric absorbs more energy before it undergoes structural changes viz., melting the crystalline regions (cf. Figure 8a,b). Moreover that, the peak height of the dyed fabric (~ − 3.8 mW) is higher than that of the blank one (~ − 0.5 mW) which indicates the strong heat absorption^68^. Further investigation in the DSC chart of the dyed wool fabric shows a weak exothermic peak with (∆H ~ − 15 J/g and peak height~ − 3.5mW), along with the slow rate of decomposition at higher temperature in the TGA chart compared to the blank wool fabric, related to the stable protective char formation as a result of reaction of the fabric with the rice straw extract (RSE)^69^. Finally, the strong absorption peak at 440 ℃ of ∆H ~ 65.2 J/g proved the higher stability of the fabric after treatment as the fabric needed to absorb such a high energy for its decomposition.

Similarly, the DSC chart of nylon 6 fabric shown in Figure 9c,d declares a high thermal stability by increasing the heat absorption peaks of ∆H ~ 105.7 and 15.7 J/g to ~ 230 and 93 J/g. The increase in the absorption heat ∆H means that the degree of crystallinity of the fabrics may have increased after treatment (*as declared by the CI% results in Sect.  3.9*.).

Also, the strong exothermic peak in the blank nylon 6 fabric (∆H~ − 33 J/g and peak height ~ − 5mW), disappeared after treatment which emphasized that the treatment decreased the combustion energy release and promoted stable protective char^70^. The char peak resulted from combustion was also affected by the antioxidant action of the extract where the radical trapping action of the material reduces that combustion energy release^71^ and hence decreases or eliminates the exothermic peak.

Thermal stability of the fabrics shown in TGA charts (cf. Fig. 7) that was represented in the decrease in the weight loss and increase in the char residual weight compared to the blank fabrics, and the increase in the heat capacity of the dyed fabrics shown in DSC charts (cf. Fig. 7) suggested that the rice straw extract (RSE) is a potential candidate as a flame retardant material for the proteinic and synthetic fabrics^72^.

Additionally, it was observed that the dehydration of the dyed wool fabric exhibited in TGA chart represented by the decrease in the initial loss in weight that also suggests that RSE is a related flame-retardant material for the used fabrics^73^.

## Conclusion

In this study, rice straw was successfully valorized through the extraction of phenolic rich compounds that were identified and quantified by LC-MS/Ms analysis. Several phenolic acids and flavonoids were detected in the rice straw extract (RSE), including p-Coumaric acid, Ferulic acid, Caffeic acid, Syringic acid, Vanillin, Chlorogenic acid, 3,4-Dihydroxybenzoic acid, hesperetin, kaempferol, and methyl gallate. RSE was used for dyeing and finishing wool and silk, as proteinic natural fabrics, and nylon 6 as synthetic fabrics under different reaction conditions. The dyed fabrics exhibited brilliant color from beige to brown with good fastness properties which confirm the potential of RSE as a natural dye. In addition, the dyed fabrics displayed high antioxidant activity with improved UV resistance compared to untreated ones. Moreover, the thermal data revealed improved thermal stability of treated fabrics, suggesting a potential contribution to enhanced thermal resistance. From an application perspective the utilization of rice straw as low cost and abundant agricultural waste provides an eco-friendly and economically attractive approach for multifunctional textile finishing. Nevertheless, further studies addressing scalability long-term durability and optimization for different fiber types are required to facilitate industrial implementations.

## Data Availability

All data is provided within the manuscript.
